# iTRAQ-Based Quantitative Proteomics Identifies Potential Regulatory Proteins Involved in Chicken Eggshell Brownness

**DOI:** 10.1371/journal.pone.0168750

**Published:** 2016-12-22

**Authors:** Guangqi Li, Congjiao Sun, Guiqin Wu, Fengying Shi, Aiqiao Liu, Ning Yang

**Affiliations:** 1 National Engineering Laboratory for Animal Breeding and MOA Key Laboratory of Animal Genetics and Breeding, College of Animal Science and Technology, China Agricultural University, Beijing, China; 2 Beijing Engineering Research Center of Layer, Beijing, China; Universidad Nacional de Rosario, ARGENTINA

## Abstract

Brown eggs are popular in many countries and consumers regard eggshell brownness as an important indicator of egg quality. However, the potential regulatory proteins and detailed molecular mechanisms regulating eggshell brownness have yet to be clearly defined. In the present study, we performed quantitative proteomics analysis with iTRAQ technology in the shell gland epithelium of hens laying dark and light brown eggs to investigate the candidate proteins and molecular mechanisms underlying variation in chicken eggshell brownness. The results indicated 147 differentially expressed proteins between these two groups, among which 65 and 82 proteins were significantly up-regulated in the light and dark groups, respectively. Functional analysis indicated that in the light group, the down-regulated iron-sulfur cluster assembly protein (Iba57) would decrease the synthesis of protoporphyrin IX; furthermore, the up-regulated protein solute carrier family 25 (mitochondrial carrier; adenine nucleotide translocator), member 5 (SLC25A5) and down-regulated translocator protein (TSPO) would lead to increased amounts of protoporphyrin IX transported into the mitochondria matrix to form heme with iron, which is supplied by ovotransferrin protein (TF). In other words, chickens from the light group produce less protoporphyrin IX, which is mainly used for heme synthesis. Therefore, the exported protoporphyrin IX available for eggshell deposition and brownness is reduced in the light group. The current study provides valuable information to elucidate variation of chicken eggshell brownness, and demonstrates the feasibility and sensitivity of iTRAQ-based quantitative proteomics analysis in providing useful insights into the molecular mechanisms underlying brown eggshell pigmentation.

## Introduction

Brown eggs dominate commercial markets in many countries, such as China, Britain, France, Italy and Portugal. Eggshell color has low genetic correlation with external and internal egg quality traits [1]. However, consumers still regard eggshell color as an important indicator of egg quality. Market tests suggest that consumers positively perceive brown eggs as being more nutritious and flavorful, and that hens laying brown eggs are thought to be organically fed [2]. Consumers who prefer brown eggs also pay attention to the intensity of eggshell brownness, and exhibit a preference for uniformity in eggshell brownness [3].

Protoporphyrin IX, an iron-free form of heme, is one of the commonest natural sources of porphyrin and the dominant pigment in brown eggshell [4, 5]. Porphyrin and its derivatives, originally called the coloring of life, play an important role in life living organisms [6]. Porphyrin contains four pyrrole rings (tetrapyrrole)connected by methane groups and there are fifteen kinds of possible isomers formed by the side chains on the porphyrin rings through different combinations [3, 7]. The structure of tetrapyrrole is highly conjugated which produces absorption bands in the visible region. Thus, eggs with protoporphyrin IX deposited onto the shell exterior will appear brown. Protoporphyrin IX destined for the avian eggshell is synthesized in the shell gland [8–11] and accumulates in the epithelial cells progressively after ovulation. It is secreted into the uterine fluid in the final stage of eggshell formation; therefore, protoporphyrin IX is mainly found in the cuticle of the eggshell [12]. Low levels of protoporphyrin IX are observed in the epithelial cells of the shell gland when the egg is in the isthmus [13] and amounts of protoporphyrin IX are maximal 3–4 hours before oviposition [14, 15]. There are three kinds of secretory cells in the chicken shell gland, including ciliated and non-ciliated columnar cells in the shell gland epithelium and tubular gland cells in the mucosa. These cells secret proteinaceous and acid mucopolysaccharide material during eggshell mineralization and are active in the formation of the shell cuticle [16]. Protoporphyrin IX is accumulated and secreted only by the ciliated apical cells in the shell gland epithelium throughout the ovulation cycle [13, 15, 17].

Studies since the 1920’s have consistently shown that the color in brown eggshells was controlled by several genes [18, 19]. However, very few additional studies have been conducted to investigate the responsible genes which regulate the intensity of eggshell brownness; moreover, the genetic mechanisms remain to be clearly defined. Proteomic analysis is a major platform for discovering functional genes which regulate biological function at the protein level. Herein, we set out to analyze the shell gland epithelium proteome from hens laying dark and light brown eggs using isobaric tags for relative and absolute quantitation (iTRAQ), a powerful technology to identify and measure the expression levels of relevant sets of proteins [20], in order to gain insight into the molecular mechanisms underlying the variation of eggshell brownness.

## Materials and Methods

### Chickens and sample collection

Two hundred and eighty-one pure-line hens laying brown eggs were used for the study. All the experimental chickens were raised in individual cages and fed with a commercial layer diet. The entire procedure was strictly performed according to the protocol approved by the Animal Welfare Committee of China Agricultural University (Permit number XK622). At 28 wk of age, eggs were collected on 3 successive days to measure the eggshell color (ESC), eggshell weight (ESW), eggshell strength (ESS), eggshell thickness (EST) and egg shape index (ESI). Each eggshell trait phenotype value was represented by the average value of the three eggs. The eggshell color was measured on the L*a*b* color space system using a reflectometer (Konica Minolta, Tokyo, Japan). Eggshell weight was measured using an electronic scale with an accuracy of 0.1 g (Mettler Toledo, Zurich, Switzerland). Eggshell strength was measured vertically with the eggshell force gauge (Model-II, Robotmation, Tokyo, Japan). Eggshell thickness was measured with digital micrometer (Mitutoyo, Japan). Egg shape index is the ratio of egg length and egg width measured by an Egg Shape Index Gauge (FHK, Tokyo, Japan, 1 mm).

**Fig 1:**
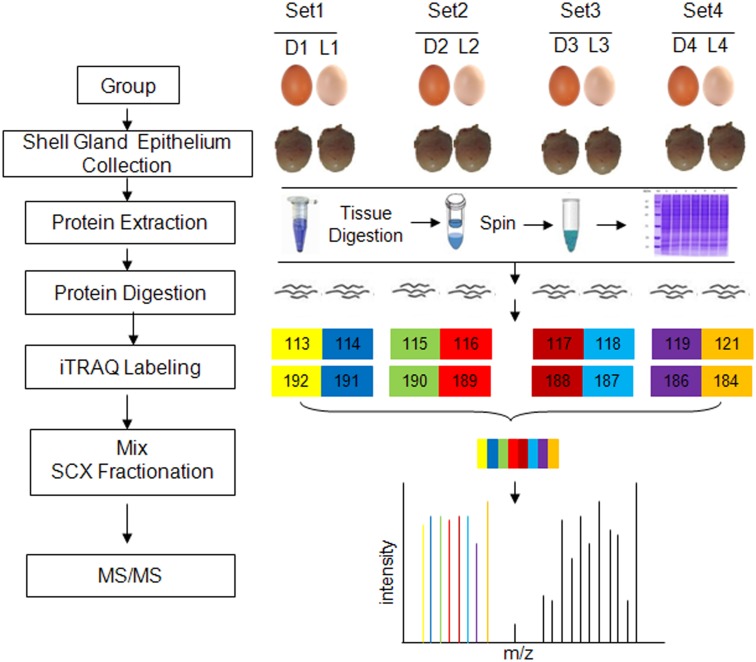
Proteomic workflow for shell gland from chickens laying dark and light brown eggs.

A total of 15 hens laying dark brown eggs and 15 hens laying light brown eggs were selected according to eggshell color and other eggshell qualities. Eggs from the two groups differed significantly in eggshell color, but not in eggshell weight, eggshell strength, eggshell thickness or egg shape index. The oviposition time of these 30 selected chickens (28–29 wks of age) was recorded at hourly intervals from 06:00 am to 14:00 pm each day. Four chickens laying dark brown eggs (D) and four chickens laying light brown eggs (L), with fairly uniform oviposition time, were slaughtered 6 h after oviposition; the entire shell gland epithelium was collected and frozen in liquid nitrogen immediately to be used for iTRAQ analysis (Fig 1) and Western blotting study.

### Protein extraction and digestion

Protein was extracted from each shell gland using a Total Protein Extraction Kit (CWBIO, Beijing, China) according to the manufacturers’ instructions and kept at −80°C for iTRAQ and Western blotting assays. The proteins concentration and quality were determined with a Protein Assay Kit (Bio-Rad, Hercules, CA, USA) and confirmed by SDS-PAGE on a 12% gel (Geneview, USA). Disulfide bonds were reduced in supernatant proteins by treating protein (100μg) with 10 mM DTT, followed by alkylation with 55 mM iodoacetamide [21]. Tryptic digestion was performed with Trypsin Gold (Promega, Madison, WI, USA) at a ratio 30: 1(protein to trypsin) at 37°C for 16 hours.

### iTRAQ labeling and SCX fractionation

After tryptic digestion, peptides were dried by vacuum centrifugation and then reconstituted in 0.5M TEAB and processed according to the manufacture’s protocol with 8-plexiTRAQ reagent (Applied biosystems, Foster City, CA) [22, 23]. Briefly, one unit of iTRAQ reagent was thawed and reconstituted in 24 μL isopropanol. The peptides were labeled with the isobaric tags and incubated at room temperature for 2h. The labeled peptide mixtures were then pooled and dried by vacuum centrifugation. The SCX chromatography of the iTRAQ labeled peptide mixtures was performed with a LC-20AB HPLC Pump system (Shimadzu, Kyoto, Japan) after reconstitution and elution with a 4.6×250 mm Ultremex SCX column containing 5μm particles (Phenomenex) at 214 nm. The collected fractions were desalted with a Strata XC18 column (Phenomenex) and vacuum-dried for further analysis.

### LC-ESI-MS/MS analysis

The collected fractions were resuspended in a mixed solution (5% ACN, 0.1%FA) and centrifuged at 20,000 xg for 10 minutes. A sample of the supernatant (5μl) was loaded with an autosampler onto a LC-20AD nanoHPLC (Shimadzu, Kyoto, Japan) with a 2 cm C18 trap column; peptides were separated on a 10 cm analytical C18 column (inner diameter 75 μm) that was packed in-house. Data acquisition was performed with a TripleTOF 5600 System (AB SCIEX, Concord, ON) [21] fitted with a Nanospray III source (AB SCIEX, Concord, ON) and a pulled quartz tip as the emitter (New Objectives, Woburn, MA).

### Proteome data analysis

Raw data files were converted into MGF files using Proteome Discoverer 1.2 (PD 1.2, Thermo) and the MS/MS spectra were analyzed using the Mascot search engine (Matrix Science, London, UK, version 2.3.02) [24] to query the Ensembl (gallus_gallus4) database(ftp://ftp.ensembl.org/pub/release-76/fasta/gallus_gallus/pep/) containing 55387 sequences. The peptide masses and fragment ion tolerances were permitted to within ± 0.05Da and ± 0.1Da, respectively, with allowance for one missed cleavage in the trypsin digests. Other search parameter settings were as follows: Gln—> pyro-Glu (N-term Q), Oxidation (M), Deamidation (NQ) as the potential variable modifications, and Carbamidomethyl (C), iTRAQ8plex (N-term), iTRAQ8plex (K) as fixed modifications. To reduce the probability of false peptide identification, only peptides at the 95% confidence interval by a Mascot probability analysis greater than “identity” were counted as identified. Each confident protein identification was based on at least one unique peptide. Proteins containing at least two unique peptides were quantified and the proteins with a 1.2-fold change between dark group and light group and a *P*-value of statistical evaluation less than 0.05 were determined as differentially expressed proteins. The annotation analysis of differentially expressed proteins was conducted by the Blast2GO software, compared with the genome background, to obtain all significantly enriched GO terms.

### Validation of differentially expressed proteins by Western blot

Western blot analysis was used for detection of ovotransferrin abundance in the two groups. Briefly, protein samples (10 ug) from the same shell gland tissues used in the proteomic analysis were prepared in SDS sample buffer and then separated on 10% SDS-PAGE gel, followed by electrophoretic transfer onto polyvinylidene fluoride (PVDF) membrane (Millipore Inc. MA, USA). The membranes were blocked in TBST with 5% nonfatmilk for 2 h at room temperature and then incubated for 24 h with primary antibodies, rabbit anti-ovotransferrin polyclonal antibody (0.5 μg/ml, EasySee, Beijing, China) and anti-GAPDH antibody (1:10000, EasySee, Beijing, China). The membranes were washed three times with TBST and subsequently incubated with goat anti-rabbit IgG (H+L), HRP Conjugate(1:5000, EasySee, Beijing, China) for 1 h at room temperature. Finally, the signals were detected using Clarity-Enhanced Chemiluminescence (ECL) reagent (Thermo Scientific) and analyzed with Quantity One software. To compare band intensity, the mean intensity from an identical area around each band was found and subtracted from a “blank” area on the same blot. Data shown is the band intensity normalized to the GAPDH controls in each lane.

## Results

### Comparison of eggshell quality between the two groups

A total of 765 eggs from 281 hens were collected on three consecutive days, and eggshell color, egg shape index, eggshell strength, eggshell thickness and eggshell weight were measured. The L* value was used to represent the intensity of eggshell brownness, as verified by the high correlation coefficient between the L*value and protoporphyrin IX quantity in the eggshell [11]. Lower L* values are associated with darker eggshells, and vice-versa. The Shapiro-Wilk normality test showed that the eggshell color (L* value) of the 281 hens followed a normal distribution (W = 0.9938, p-value = 0.3077). Chickens were selected according to L* values from two tails of the selected eggs; the selected chickens consistently laid eggs with uniform eggshell brownness throughout this laying period. Although the two groups of chickens used for proteomic analysis laid eggs differing significantly in eggshell color, they did not differ in other egg parameters (egg shape index, eggshell strength, eggshell thickness and eggshell weight) (Table 1). This sample selection method was applied in order to eliminate potential interference caused by differences in the other eggshell traits.

**Table 1:** Comparison of eggshell quality between the two groups.

Eggshell quality	Dark group (N = 4)	Light group (N = 4)	Population (N = 281)
Eggshell color (L*)	55.47±0.65^C^	69.82±0.43^A^	62.83±3.43^B^
Eggshell color (a*)	20.94±0.48^A^	13.42±0.48^C^	17.20±1.87^B^
Eggshell color (b*)	30.27±1.08^A^	24.22±2.40^B^	29.12±1.90^A^
**Eggshell weight (g)**	5.54±0.34	5.46±0.38	5.75±0.51
**Eggshell strength(kg/cm**^**2**^**)**	3.623±0.7011	3.629±1.029	3.752±0.75
**Eggshell thickness (mm**)	0.316±0.015	0.311±0.009	0.313±0.023
**Egg shape index**	1.28±0.05	1.28±0.02	1.27±0.07

^A/B/C^means with different superscripts within each row differ significantly (*P*<0.01).

N = Number of hens.

### Identification of differentially expressed proteins

The objective of the present study was to identify proteomic differences in the shell gland epithelium of chickens laying light and dark brown eggs in order to provide insight into the mechanisms regulating the intensity of eggshell brownness. Four different biological replicates from each group were analyzed in order to estimate the relative abundance of proteins using iTRAQ-labeling technology.

**Fig 2:**
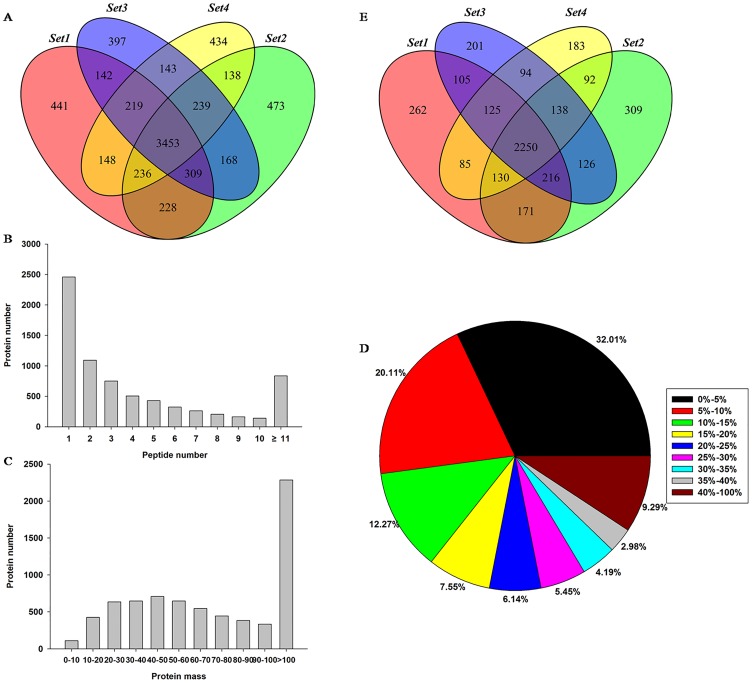
Results of the proteome analysis. A: Venn diagrams showed the overlap of identified proteins in the four biological replicates, which were expressed as set 1, set 2, set 3 and set 4. B: The peptide number distribution of identified proteins. C: The distribution of identified proteins mass. D: Pie charts showing the distribution of protein coverage. E: Venn diagrams showed the overlap of quantified proteins in the four biological replicates, which were expressed as set 1, set 2, set 3, and set 4.

The iTRAQ data showed that a total of 64155, 68823, 61687and 60035 MS/MS spectra in the four biological replicate sets (set1, set2, set3 and set4) could be matched to known spectra by the Mascot software (version 2.3.02); of these, 54400, 57965, 51924 and 50697 unique spectra were matched to 28714, 30229, 27897 and 26246 peptides, respectively. After data filtering, 25342, 26584, 24554 and 23126 unique peptides remained, which permitted identification of 5176, 5244, 5070 and 5010 proteins in the four sets (Table 2). Among the identified proteins, 3453 were common to all four biological replicates (Fig 2A). Most proteins were identified by fewer than ten peptides (Fig 2B) and the masses of identified protein were distributed from 10 to 100 kDa with good average coverage (Fig 2C and 2D). The abundance of proteins identified by at least two unique peptides was measured, and a total of 3344, 3432, 3255 and 3097 proteins were reliably quantified in the four biological replicates, among which, 2250 proteins were common to all four biological replicates (Fig 2E and S1 Table). Of the 2250 common proteins, 147proteins with a fold change > 1.2 and a *p* value < 0.05, in at least two replicates and simultaneously present in the other two replicates, conformed to this variation trend and were determined to be differentially expressed proteins. These included 65 significantly up-regulated proteins and 82 significantly down-regulated proteins (S2 Table).

**Table 2:** Descriptive statistics for proteomic identification in the four sets.

Set	Total Spectra	Spectra	Unique Spectra	Peptide	Unique Peptide	Identified Protein	Quantified Protein	Up Proteins	Down Proteins
**Set1**	384327	64155	54400	28714	25342	5176	3344	44	41
**Set2**	389313	68823	57965	30229	26584	5244	3432	49	53
**Set3**	411613	61687	51924	27897	24554	5070	3255	58	53
**Set4**	374440	60035	50697	26246	23126	5010	3097	40	54

Up Proteins = Number of significantly up-regulated proteins, fold change > 1.2, *P*<0.05. Down Proteins = Number of significantly down-regulated proteins, fold change < 0.84, *P*<0.05.

### Classification and pathway analysis of differentially expressed proteins in S2 Table

**Fig 3:**
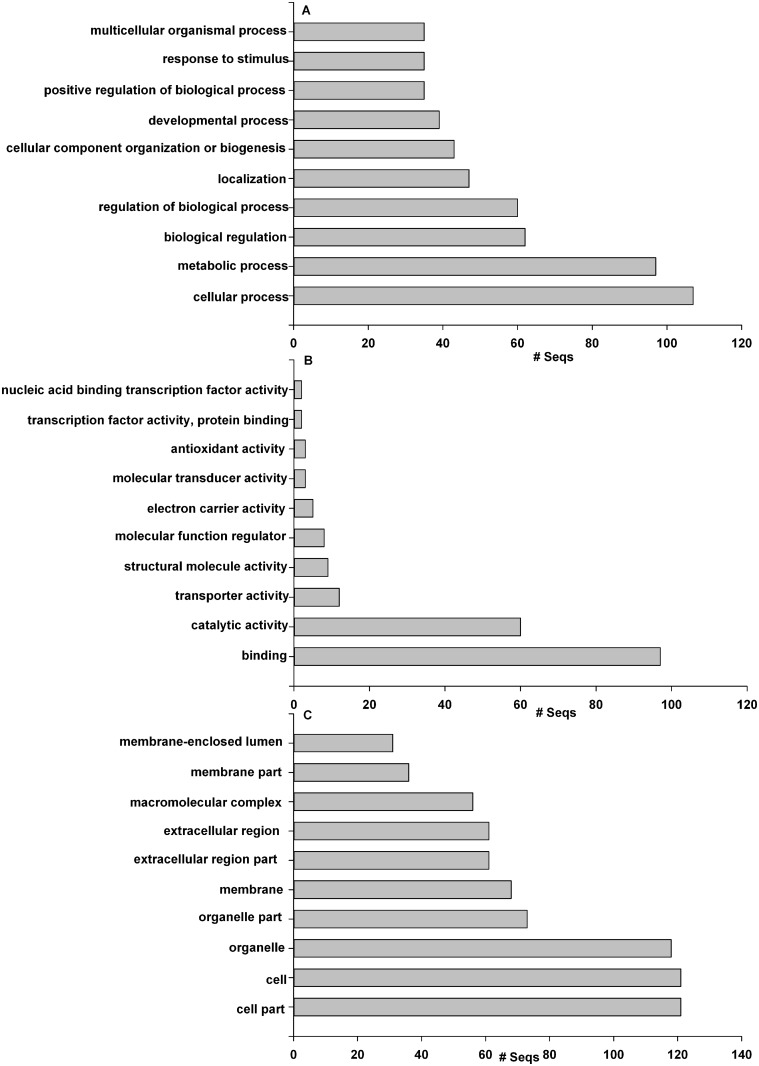
(A–C) Charts showing the functional categorization of genes with significant protein expression differences between dark group and light group. The proteins were categorized based on GO annotation, and the number of each category is displayed based on (A) biological process, (B) molecular function and (C) cellular components.

To identify proteins and signaling pathways that are vital to regulate function in the synthesis, transport, accumulation and deposition of protoporphyrin IX, functional analysis of the differentially expressed proteins in S2 Table was carried out using Blast2GO software (version 3.2). Proteins were categorized according to their cellular component (CC), molecular function (MF) and biological process (BP) (Fig 3). The cellular component annotation revealed that the significantly changed proteins were involved in cell part(GO:0044464), organelle (GO:0043226), membrane (GO:0016020) and extracellular region (GO:0005576). Molecular functions of the annotated proteins were mainly associated with three GO terms. The term of binding (GO: 0005488) contains several sub-terms including protein binding (GO: 0005515), ion binding (GO: 0043167), cofactor binding (GO: 0048037), organic cyclic compound binding (GO: 009715), heterocyclic compound binding (GO: 1901363). The term of catalytic activity (GO: 0003824) encompasses biologically catalyzed reactions involving substrates and enzymes, which occurs at physiological temperatures. Transporter activity (GO: 0005215) involves small-molecule carriers or transporters, which enables the intracellular movement or intercellular transport of macromolecules, small molecules and ions. The biological process annotation revealed that the significantly changed proteins were involved in four classes of GO terms. The cellular process (GO: 0009987) is signal conversion in which a signal is conveyed to trigger changes at the cellular level. The metabolic process (GO: 0008152) are chemical reactions and pathways including small molecules transformation, DNA repair and replication, and protein synthesis and degradation. The term of biological regulation (GO: 0065007) describes modulation of a measurable attribute in regulation of biological process, biological quality and molecular function. Localization (GO: 0051179) is the location, establishment and maintenance of substance or cellular components, such as macromolecule localization, nitric oxide storage and localization of cell.

Pathway analysis of the differentially expressed proteins in S2 Table using Blast2GO software (version 3.2) revealed enrichment of most proteins was involved in four pathways (Table 3). The purine metabolism pathway (map00230) involves purine synthesis and breakdown, which is carried out in many organisms. In the thiamine metabolism pathway (map00730), glycine is the basic substrate, which is also the key substrate in the first step of protoporphyrin IX synthesis. Oxidative phosphorylation (map00190) is an energy-releasing chemical reaction in which nutrients are oxidized by enzymes and energy is released by transferring electrons to oxygen. In our study, four differentially expressed proteins which are involved in this pathway were all up-regulated in the light group. The citrate cycle (TCA cycle, map00020), also called the tricarboxylic acid cycle, involves in a series of enzyme-catalyzed chemical reactions which occurs in the mitochondrial matrix.

**Table 3:** Main pathways of differentially expressed proteins in S2 Table.

Pathway	^#^ Proteins	Protein ID	Gene Symbol	Fold Change (L/D)	Map ID
**Purine metabolism**	11	ENSGALP00000002236	RAP1A	1.39	map00230
		ENSGALP00000033584	CRHOA	1.24	
		ENSGALP00000024757	RBF	1.26	
		ENSGALP00000002695	ATP5A1	1.85	
		ENSGALP00000000062	Novel	0.74	
		ENSGALP00000013046	ARL3	1.3	
		ENSGALP00000038795	ATP5A1W	1.78	
		ENSGALP00000010899	ATP5C1	1.4	
		ENSGALP00000002205	ATP5F1	1.23	
		ENSGALP00000008546	GUK1	1.45	
		ENSGALP00000008095	ADK	1.32	
**Thiamine metabolism**	11	ENSGALP00000015909	RAB33B	0.76	map00730
		ENSGALP00000001352	CGN	0.78	
		ENSGALP00000002236	RAP1A	1.39	
		ENSGALP00000033584	CRHOA	1.24	
		ENSGALP00000024757	RBF	1.26	
		ENSGALP00000002695	ATP5A1	1.85	
		ENSGALP00000000062	Novel	0.74	
		ENSGALP00000013046	ARL3	1.3	
		ENSGALP00000010899	ATP5C1	1.4	
		ENSGALP00000002205	ATP5F1	1.23	
		ENSGALP00000023849	ATP6V1A	0.79	
**Oxidative phosphorylation**	4	ENSGALP00000042172	COX7A2L	1.32	map00190
		ENSGALP00000014321	NDUFA5	1.33	
		ENSGALP00000034622	ND5	1.66	
		ENSGALP00000000693	Novel	1.28	
**Citrate cycle (TCA cycle)**	3	ENSGALP00000028857	OGDH	1.6	map00020
		ENSGALP00000000693	Novel	1.28	
		ENSGALP00000015626	PCK2	0.76	

^#^ Proteins = Number of proteins involved in the pathway.

### Verification of ovotransferrin expression by Western blot

**Fig 4:**
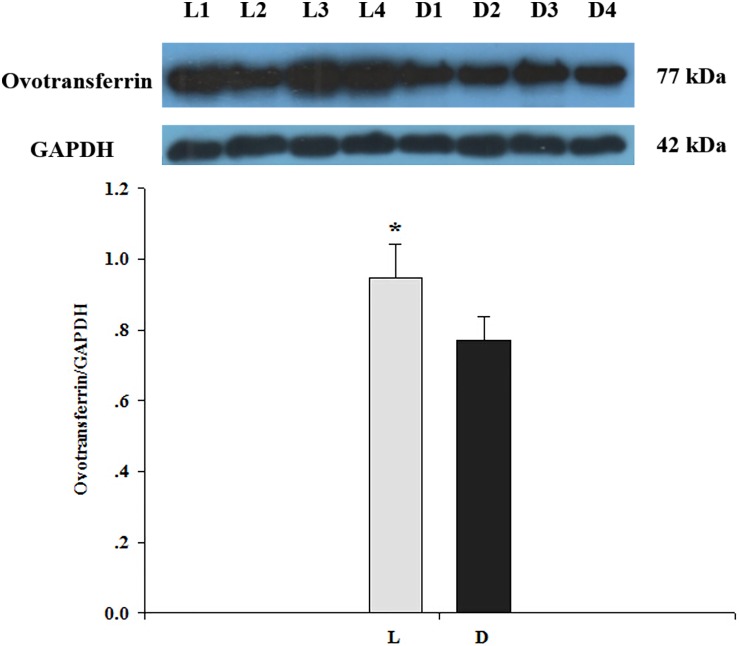
Analysis of ovotransferrin abundance in the two groups detected by Western blot. Proteins id are indicated above the figures. The protein names are listed on the left of the figure and the positions of molecular weight (kDa) are indicated to the right of the figure. Values are means ± SD;* p< 0.05 (n = 4).

To confirm the results of the differentially expressed proteins identified by iTRAQ analysis (S2 Table), Western blotting was performed to detect the levels of ovotransferrin in the two groups. GAPDH was monitored as an internal control. The Western blot results showed that the levels of ovotransferrin in the shell gland epithelium of the light group were higher (Fig 4), which is in agreement with the results of iTRAQ analysis.

## Discussion

Proteomics is a valuable approach which has been useful for detecting and quantifying chicken proteins associated with disease resistance traits and eggshell mechanical properties [25–27]. Isobaric tags for relative and absolute quantification (iTRAQ) analysis can greatly increase the identification sensitivity and quantitative accuracy of proteomic analyses through a multiplexed quantitative strategy; it is a powerful tool for quantitative proteomic analysis that has been widely applied in several studies [28–30]. In the current study, we have used the iTRAQ technology for the first time to identify the differentially expressed proteins in the shell gland epithelium of chickens that lay eggs with distinct levels of brownness.

The intensity of eggshell brownness is dependent on the concentration of protoporphyrin IX in the eggshell [11], which will be affected by the total amount of protoporphyrin IX and the size of eggshell. Given the same amount of protoporphyrin IX, larger eggs will have lighter brownness than smaller eggs. To avoid this interference, we identified two groups of hens that laid eggs with different levels of eggshell brownness, but with similar egg shape index, eggshell thickness and eggshell weight. This approach ensured that the difference in brownness between two groups was caused by the total amount of protoporphyrin IX, rather than the size of eggshell.

The results showed that 2250 proteins were reliably identified in the chicken shell gland epithelium, and 147 proteins were differentially expressed between the two groups. The biosynthesis, transport, accumulation and deposition of protoporphyrin IX in the shell gland are coordinated with eggshell mineralization. At 6 h post-oviposition the next egg reaches the shell gland to undergo mineralization during nearly 19 hours; meanwhile, the protoporphyrin IX granules can be observed in the epithelium of the shell gland, suggesting that the proteins associated with protoporphyrin IX synthesis and transport are active at this time. Our previous studies indicated that the intensity of eggshell brownness was determined by the content of protoporphyrin IX deposited onto the eggshell, and that the protoporphyrin IX content in the eggshell depends on the accumulation of protoporphyrin IX in the shell gland epithelium [11]. Therefore, the differentially expressed proteins (S2 Table) that are involved in protoporphyrin IX synthesis and transport warrant further investigation. The biosynthesis pathway for protoporphyrin IX has been fully elucidated. Protoporphyrin IX is formed in the mitochondrion and the involved enzymes are mainly located in the mitochondrial inner or outer membrane, suggesting that the differentially expressed proteins located in the mitochondrial inner or outer membrane, based on their cellular component, might be active during the synthesis and transport of protoporphyrin IX. The functional and pathway analysis gave an overall view of the differentially expressed proteins (S2 Table). In addition, we searched databases and related literature for detailed functional descriptions of these differentially expressed proteins. Finally, we identified eight putative protein candidates (Table 4). These include four proteins that could function in protoporphyrin IX synthesis and transport: iron-sulfur cluster assembly protein (Iba57), ovotransferrin (TF), the 18 kDa translocator protein (TSPO) and the adenine nucleotide transporter (SLC25A5). An additional four proteins are involved in the oxidative phosphorylation pathway and could play vital roles in the production and accumulation of protoporphyrin IX in the shell gland epithelium: cytochrome c oxidase subunit (COX7A2L), NADH dehydrogenase (NDUFA5), NADH-ubiquinone oxidoreductase chain 5 (ND5) and succinate dehydrogenase.

**Table 4:** Putative proteins influencing chicken eggshell brownness.

Protein ID	Protein Description	Gene Symbol	Fold Change (L/D)	Function Description
**ENSGALP00000010405**	Ovotransferrin	TF	1.509	iron-transfer and protective activities
**ENSGALP00000008534**	Chromosome 1 open reading frame 69	Iba57	0.605	Catalyze the maturation of mitochondria [4Fe-4S] proteins
**ENSGALP00000022870**	Translocator protein	TSPO	0.711	Export protoporphyrin IX out of mitochondria
**ENSGALP00000014105**	Solute carrier family 25 (mitochondrial carrier; adenine nucleotide translocator), member 5	SLC25A5	1.250	Import protoporphyrin IX into mitochondria matrix
**ENSGALP00000042172**	Cytochrome c oxidase subunit VIIa polypeptide 2 like	COX7A2L	1.32	Catalyzes the electron transfer from reduced cytochrome c to oxygen
**ENSGALP00000014321**	NADH dehydrogenase (ubiquinone) 1 alpha subcomplex, 5	NDUFA5	1.33	Transfers electrons from NADH to ubiquinone
**ENSGALP00000034622**	NADH-ubiquinone oxidoreductase chain 5	ND5	1.66	Transfer of electrons from NADH to the respiratory chain
**ENSGALP00000000693**	Succinate dehydrogenase	Novel	1.28	Catalyzes the oxidation of succinate to fumarate with the reduction of ubiquinone to ubiquinol

Iba57 is a novel member of the mitochondrial ISC assembly system, which belongs to the COG0354 protein family and occurs in all organisms. Iba57 is located in mitochondrial matrix and plays specific roles in the maturation of mitochondria [4Fe-4S] proteins [31–33]. The [4Fe-4S] clusters are essential cofactors for the activation of numerous key mitochondrial enzymes including aconitase and lipoic acid synthase [32, 34, 35]. The latter enzyme generates lipoate, a cofactor for the function of pyruvate dehydrogenase (PDH) enzymes to convert pyruvate into acetyl-CoA, which enters the citric acid cycle to form citrate. Aconitase converts citrate to isocitrate [36], which leads to synthesis of succinyl-CoA [37], a substrate in the first and rate-limiting step of heme synthesis. In situ hybridization analysis using zebrafish embryos at 24 hour post-fertilization shows that Iba57 is specifically expressed in the intermediate cell mass which is functionally equivalent to the hematopoietic tissues; knockdown of Iba57 results in reduction of heme production, and thus causes severe anemia in zebrafish [38]. It is hypothesized that Iba57 provides [4Fe-4S] protein for lipoic acid synthase and aconitase, which affects the production of succinyl-CoA and regulates heme synthesis. The down-regulated Iba57protein in the light group would reduce [4Fe-4S] protein production, which in turn leads to reduced production of succinyl-CoA, and results in a decreased rate of heme formation. Protoporphyrin IX is the immediate precursor to heme. Hence, we propose that a decline in the rate of heme synthesis would diminish the production of protoporphyrin IX.

Ovotransferrin (TF) protein belongs to the iron-binding protein family and is endowed with both iron-transfer and protective activities [39]. Contrary to the mammalian genome, the avian genome contains only one transferrin gene which is expressed both in liver and oviduct [40]. The chicken ovotransferrin gene, first identified in 1944, has a size of 10.5 kb and is organized in 17 exons and 16 introns [41]. The encoded ovotransferrin protein is a 77.90 kDa glycoprotein, with the capability to reversibly bind Fe^3+^; it is involved in iron transport and homeostasis [42]. The pathway for heme synthesis is fully understood now. In the last step, ferrochelatase inserts an iron into protoporphyrin IX to produce heme [43, 44]; the rate of iron uptake from transferrin limits the rate of heme synthesis [45]. Therefore, low iron levels will result in the accumulation of protoporphyrin IX in the cell [46]. In the shell gland of the light group, the iron levels may be greater due to higher levels of ovotransferrin protein, which will promote the consumption of protoporphyrin IX to form heme. Therefore, there will be less protoporphyrin IX left to be used for eggshell pigmentation.

The 18 kDa translocator protein (TSPO), previous known as the peripheral-type benzodiazepine receptor or recognition site (PBR), was first identified in 1977 by its capability to bind benzodiazepines [47]. TSPO protein is a transmembrane protein localized primarily in the mitochondrial outer membrane and is widely and conservatively expressed in fungi, bacteria, plants and animals [48], participating in cell respiration, steroidogenesis, cell apoptosis and the transport of benzodiazepines and tetrapyrrole [49–52]. TPSO had been previously demonstrated to have high affinity with porphyrin, especially protoporphyrin IX, in bacteria, vertebrates and mammals [53, 54], and involved in heme synthesis by transporting protoporphyrin IX [52]. In cultured cells exposed to protoporphyrin IX, TSPO prevented intracellular accumulation of protoporphyrin IX [55]; leading to speculation that the role of TSPO in protoporphyrin IX transportis export. Therefore, TSPO density and distribution in cells could be a predictor of the capacity for protoporphyrin IX production [56]. In our study, the TSPO protein was down-regulated in the light group, which would decrease the rate of protoporphyrin IX export from the mitochondria; therefore, less protoporphyrin IX could be accumulated in the epithelial cell of shell gland for eggshell pigmentation. TSPO forms a ternary complex with the 32 kDa voltage-dependent anion channel (VDAC) and the 30 kDa adenine nucleotide transporter (SLC25A5) which are located in both the outer and inner mitochondrial membranes, functioning as the transport machinery for various substrates and endogenous ligands including porphyrins [57, 58]. Our results showed that the SLC25A5 protein was up-regulated in the light group compared with the dark group. The SLC25A5 protein is an inner membrane transporter that facilitates the exchange of ATP and ADP and also contributes to heme biosynthesis by serving as an alternative protoporphyrin IX transport pathway to import it into the mitochondrial matrix for heme synthesis [59, 60]. SLC25A5 gene knockout mice showed a pale phenotype and postnatal growth was severely retarded with macrocytic anemia because of maturation arrest of the erythrocyte precursor [61]. Therefore, the up-regulated SLC25A5 protein expression in the light group would cause more protoporphyrin IX to be imported into mitochondrial matrix for heme synthesis. The overall result of differential TSPO and SLC25A5 protein expression patterns between the two eggshell groups would be a reduction in available protoporphyrin IX in the lighter eggshell pigmentation group.

Oxidative phosphorylation is a metabolic pathway that produces ATP by transporting electrons to acceptors such as oxygen. Oxygen is necessary in the enzymic biosynthesis of protoporphyrin IX and the rate of protoporphyrin IX formation is enhanced by an increase in oxygen concentration near the normal range (20%, v/v) [62]. In mammals, protoporphyrinogen oxidase is the key enzyme which catalyzes the oxidation of protoporphyrinogen IX to protoporphyrin IX; however, protoporphyrinogen oxidase in birds has not yet been identified. In fact, the reaction of protoporphyrinogen IX oxidization to protoporphyrin IX is facile; in the absence of protoporphyrinogen oxidase, protoporphyrinogen IX can be auto-oxidized to protoporphyrin IX in the presence of oxygen. Sparks (2011) speculated that, in avian species, colourless protoporphyrinogen IX is synthesized in the tubular gland cells and then transported to epithelial cells of the shell gland to be auto-oxidized to protoporphyrin IX [3]. Therefore, the oxygen concentration in the shell gland cells is important for regulating the formation of protoporphyrin IX. In the present study, the four differentially expressed proteins involved in the oxidative phosphorylation pathway (Table 2) were up-regulated in the light group, indicating that the oxidative phosphorylation maybe more active in the shell gland of chickens from the light group. Therefore, the consumption of oxygen is greater in the shell gland of chickens from the light group which may decrease the rate of protoporphyrin IX formation.

**Fig 5:**
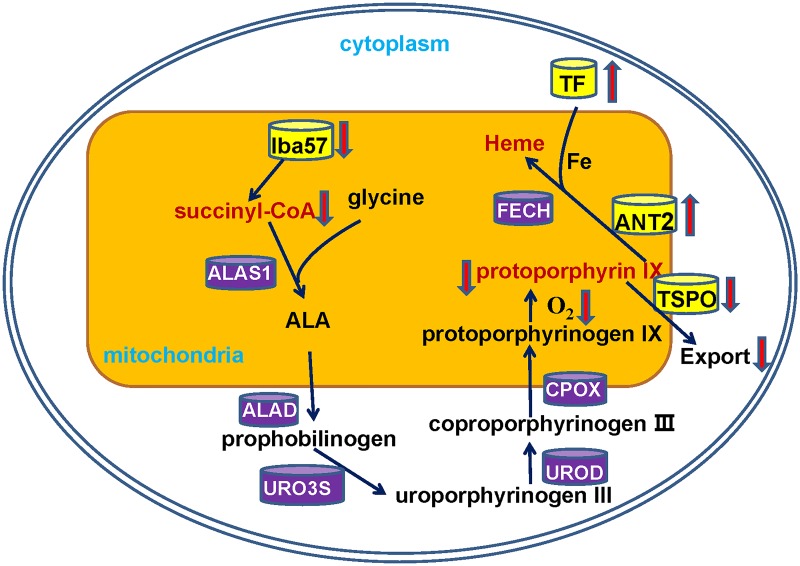
Putative interaction of candidate proteins regulating the content of protoporphyrin IX used for eggshell pigmentation. ALAS1: Delta-aminolevulinate synthase 1, ALA: Delta-aminolevulinic acid, ALAD: Delta-aminolevulinic acid dehydratase, URO3S: Uroporphyrinogen III Synthase, UROD: Uroporphyrinogen decarboxylase, CPOX: Coproporphyrinogen III oxidase, FECH: Ferrochelatase.

The protein profiling data revealed that proteins involved in the synthesis and transport of protoporphyrin IX might influence the intensity of eggshell brownness and result in different shades of eggshell brownness. It is proposed that the variation in eggshell brownness is due to the combined interactions of these proteins (Fig 5). In the shell gland of chickens from the light group, the down-regulated Iba57 protein might reduce [4Fe-4S] protein production, and then lead to the diminished production of succinyl-CoA, resulting in a decreased rate of protoporphyrin IX formation. In addition, oxidative phosphorylation was more active in the shell gland of chickens from the light shell group which would consume more oxygen and this might also decrease the rate of protoporphyrin IX formation. Moreover, up-regulated SLC25A5and down-regulated TSPO would import more protoporphyrin IX into the mitochondrial matrix where heme is synthesized. And the up-regulated ovotransferrin protein in the shell gland of chickens from the light group would transported more iron into the mitochondria, which would promote the formation of heme from protoporphyrin IX and result in the decrease of protoporphyrin IX accumulation in the shell gland epithelium cells.

## Conclusions

In summary, chickens from the light eggshell group produce less protoporphyrin IX, most of which was used for heme synthesis; therefore, the protoporphyrin IX content deposited onto the eggshell would be reduced. Proteomics is an effective tool to identify candidate proteins that regulate complex biological processes. The proteins implicated in eggshell protoporphyrin IX formation can serve as a foundation to better understand and further investigate the molecular mechanisms of eggshell pigmentation brownness.

## Supporting Information

S1 Table Protein quantification in the shell gland epithelium of hens laying light and dark eggs. (XLSX)

S2 Table Significantly altered proteins in the shell gland epithelium of hens laying light and dark eggs. (XLSX)
